# Delayed internalization and lack of recycling in a beta_2_-adrenergic receptor fused to the G protein alpha-subunit

**DOI:** 10.1186/1471-2121-9-56

**Published:** 2008-10-07

**Authors:** Maria Grazia Di Certo, Enrico M Batassa, Ida Casella, Annalucia Serafino, Aristide Floridi, Claudio Passananti, Paola Molinari, Elisabetta Mattei

**Affiliations:** 1 Istituto di Neurobiologia e Medicina Molecolare, CNR, c/o Fondazione Santa Lucia/EBRI, Via del Fosso di Fiorano 64/65, 00143 Rome, Italy; 2 Dipartimento di Biotecnologie Cellulari ed Ematologia, Sezione di Genetica Molecolare, Università di Roma "La Sapienza", Viale Regina Elena, 324 00161 Rome, Italy; 3 EBRI-European Brain Research Institute, Via del Fosso di Fiorano, 64/65, 00143 Rome, Italy; 4 Dipartimento del Farmaco, Istituto Superiore di Sanità, Viale Regina Elena 299, 00161 Rome, Italy; 5 Istituto di Neurobiologia e Medicina Molecolare, CNR, Tor Vergata, Via del Fosso del Cavaliere, 100 00133 Rome, Italy; 6 Dipartimento di Medicina Sperimentale, Via Vetoio, Coppito 2, Università de L'Aquila, 67100 L'Aquila, Italy; 7 Laboratory "B", Regina Elena Cancer Institute, Via delle Messi d'Oro, 156 00158 Rome, Italy; 8 Istituto di Biologia e Patologia Molecolari, CNR, c/o Regina Elena Cancer Institute, Via delle Messi d'Oro 156, 00158 Rome, Italy; 9 AIRC-Rome Oncogenomic Center (ROC), Via delle Messi d'Oro, 156 00158 Rome, Italy

## Abstract

**Background:**

Chimeric proteins obtained by the fusion of a G protein-coupled receptor (GPCR) sequence to the N-terminus of the G protein α-subunit have been extensively used to investigate several aspects of GPCR signalling. Although both the receptor and the G protein generally maintain a fully functional state in such polypeptides, original observations made using a chimera between the β_2_-adrenergic receptor (β_2_AR) and Gα_s _indicated that the fusion to the α-subunit resulted in a marked reduction of receptor desensitization and down-regulation. To further investigate this phenomenon, we have compared the rates of internalization and recycling between wild-type and Gα_s_-fused β_2_AR.

**Results:**

The rate of agonist-induced internalization, measured as the disappearance of cell surface immunofluorescence in HEK293 cells permanently expressing N-terminus tagged receptors, was reduced three-fold by receptor-G protein fusion. However, both fused and non-fused receptors translocated to the same endocytic compartment, as determined by dual-label confocal analysis of cells co-expressing both proteins and transferrin co-localization.

Receptor recycling, determined as the reversion of surface immunofluorescence following the addition of antagonist to cells that were previously exposed to agonist, markedly differed between wild-type and fused receptors. While most of the internalized β_2_AR returned rapidly to the plasma membrane, β_2_AR-Gα_s _did not recycle, and the observed slow recovery for the fusion protein immunofluorescence was entirely accounted for by protein synthesis.

**Conclusion:**

The covalent linkage between β_2_AR and Gα_s _does not appear to alter the initial endocytic translocation of the two proteins, although there is reduced efficiency. It does, however, completely disrupt the process of receptor and G protein recycling. We conclude that the physical separation between receptor and Gα is not necessary for the transit to early endosomes, but is an essential requirement for the correct post-endocytic sorting and recycling of the two proteins.

## Background

The activity of G protein-coupled receptors (GPCRs) is regulated by a sophisticated balance between molecular mechanisms governing receptor signalling, desensitization and resensitization. An important role in the regulation of GPCRs functions is played by the agonist-mediated internalization of receptors into intracellular compartments from which they may be sorted into specific endosomal pathways [reviewed in ref. [1-3]]. Thus, the characterization of the molecular events involved in the regulation of the intracellular trafficking of receptors is a fundamental question in cell biology.

Over the past several years, the agonist-promoted internalization of β_2_-adrenergic receptor (β_2_AR), a prototypic member of the GPCR superfamily, has become the subject of intensive studies. Several investigations established that, in response to agonist stimulation, β_2_ARs undergo rapid phosphorylation by both second messenger-dependent protein kinases and G protein-coupled receptor kinases (GRKs) [3]. This event targets receptors for the binding of arrestin proteins, which sterically uncouples receptors from their cognate heterotrimeric G proteins and favours the receptors endocytosis via clathrin-coated vesicles into endosomal compartments [1,4,5]. As a consequence of this, β_2_ARs are exposed to dephosphorylation, following which receptors are recycled back to the plasma membrane surface as fully sensitized receptors [6]. Although many of the molecular mechanisms described for β_2_AR might apply equally well to other GPCRs, the diversity in receptor structures corresponds to important differences in the intracellular trafficking patterns, as well as to the functional signal transduction of distinct GPCR subtypes. For example, regarding beta-subtypes of adrenergic receptors (β_1_, β_2 _and β_3_-AR), it is known that, unlike β_2_AR, β_3_AR does not internalize in response to agonist; likewise, human β_1_AR is more resistant to agonist-mediated down-regulation and, although it uses the same endocytosis mechanism as human β_2_AR, it is sorted to different endosomal compartments [7-9]. Other GPCRs that internalize following agonist activation are targeted to lysosomes for degradation, or are retained within endosomal compartments [10-12]. Moreover, relatively little is known about the mechanisms that determine the specificity of GPCR trafficking after endocytosis. It is established that the sorting of internalized β_2_AR between recycling and degradative endocytic pathways is controlled by a protein interaction involving the distal carboxyl-terminal cytoplasmic domain of the receptor. Mutations of this sequence inhibit efficient recycling and cause the missorting of internalized receptors to lysosomes, thereby increasing the down-regulation [13].

Important advancements in the knowledge of intracellular trafficking of β_2_AR have been reached through studies that used powerful tools, such as real-time optical-analysis, to visualize the dynamics of receptor trafficking in living cells. At first, the sub-cellular redistribution of epitope-tagged β_2_AR in response to agonist activation was observed by confocal fluorescence microscopy, and then by direct visualization of β_2_AR-green fluorescent protein (GFP) chimeras in cells transiently or stably expressing the fusion protein [14-16]. Importantly, experiments employing this technical strategy have suggested that sequestered β_2_ARs undergo processing through endosomal compartments in a similar way to that observed for constitutively internalized receptors, such as the transferrin receptor [14]. After internalization, β_2_AR rapidly colocalizes with transferrin into recycling endosomal vesicles, but following prolonged exposure to agonist, it traffics through lysosomal vesicles as part of a down-regulation process [16].

Moreover, relevant information about GPCR endocytosis emerged from studies that used GFP fusion proteins to provide the opportunity not only to observe β_2_-adrenergic receptor trafficking, but also to study the functional dynamics of G proteins in real time. It was reported that, using a Gα_s_-GFP construct, stimulation of COS-1 cells with isoproterenol resulted in the movement of the Gα_s_-GFP fusion protein from the plasma membrane to the cytoplasm [17]. Moreover, Hynes et al. recently employed strategies that allowed the simultaneous imaging of the α and βγ components of heterotrimeric G proteins in live cells. They showed that Gs and β_2_AR dissociate upon agonist stimulation, internalize via different mechanisms and traffic to distinct sub-cellular localizations [18]. To further address this question, we report here a study on agonist-mediated internalization and intracellular sorting between the wild type adrenergic receptor, β_2_AR, compared to that of the chimeric receptor, β_2_AR-Gα_s_, resulting from the fusion of β_2_-adrenergic receptor with the amino-terminal of the G protein α-subunit [19].

Although the β_2_AR-Gα_s _exhibits an unaffected ability to transduce adrenergic signals and an increased sensitivity to agonists as compared to wild type receptor, here we show that the fusion of β_2_AR to Gα_s _slows down agonist-induced internalization and strongly affects the recycling of receptor to the plasma membrane, even if β_2_AR-Gα_s _is targeted into endocytic compartments similar to those observed for the non-fused receptor.

## Results and discussion

### MAPKs activation by β_2_AR-Gα_s _in HEK293 cells

**Figure 1:**
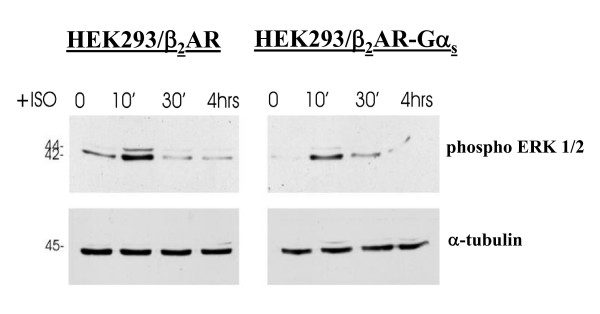
**Agonist-mediated activation of endogenous MAP kinases**. Agonist-mediated activation of MAP kinases (ERK1/2) was detected by immunoblotting performed on stably transfected cells expressing the fusion protein (HEK293/β_2_AR-Gα_s_) or the wild type β_2_-adrenergic receptor (HEK293/β_2_AR). Cells were first incubated in the absence (0) or presence of 1 μM isoproterenol (ISO) for the indicated times, and then whole-cell extracts were subjected to SDS-PAGE. The immunoreactivity of the endogenous ERK1/2 following agonist-induced phosphorylation was detected by probing blots with Phospho-p44/42 MAP kinases rabbit monoclonal antibody. Samples were normalized for protein content by reprobing blots with a monoclonal antibody to alpha-tubulin.

GPCRs activate MAPKs by multiple converging mechanisms [20,21]. To test the ability of the fusion protein β_2_AR-Gα_s _to induce MAPK signalling, the agonist-mediated activation of extra cellular signal-regulated kinases (ERK1/2), also known as p42/44 MAPK, was analysed by western blotting. HEK293 cells stably expressing the β_2_AR-Gα_s _(HEK293/β_2_AR-Gα_s_) or wild type β_2_-adrenergic receptor (HEK293/β_2_AR) were treated for increasing times with the agonist isoproterenol (1 μM), then cell extracts were subjected to SDS-PAGE and blots were probed with a monoclonal antibody specific for the phosphorylated ERK1/2 proteins. As shown in figure 1, the isoproterenol stimulation of HEK293/β_2_AR-Gα_s _resulted in both ERK-2 (major band at 42 kDa) and ERK-1 (minor band at 44 kDa) phosphorylation, whose activities were enhanced following 10 min of agonist treatment and then rapidly declined without other activation peaks within 4 hours. A similar time-course of ERK activation was observed in the cells stably expressing the wild type receptor.

In agreement with previous findings, this result confirms that the fusion of the G protein α-subunit to β_2_AR does not alter the functional properties of the receptor, which retains its ability to activate cellular signalling.

### β_2_AR-Gα_s _and β_2_AR show different internalization kinetics following agonist stimulation

**Figure 2:**
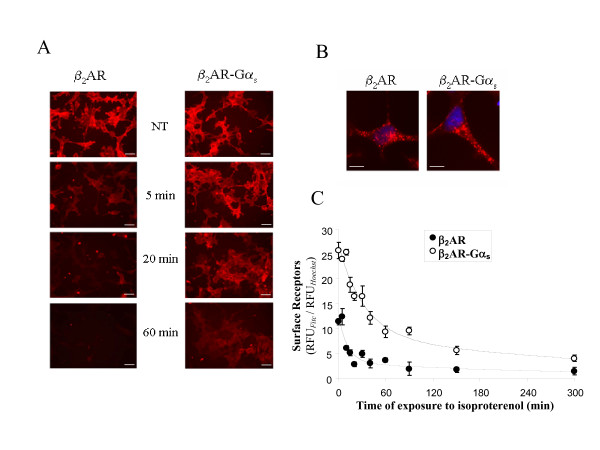
**Agonist-dependent internalization of β_2_AR-Gα_s_**. **A. **HEK293 cells stably expressing β_2_AR or β_2_AR-Gα_s _were not treated (NT) or treated with isoproterenol for 5, 20 or 60 minutes to induce receptor internalization. The cells were then fixed in 4% formaldehyde and sequentially immuno-stained with anti-Flag and Alexa-Fluor 594 conjugated anti-mouse IgG. The disappearance of the fluorescence from the plasma membrane was monitored with an epifluorescence microscope. Scale bars, 20 μm. **B. **Immunofluorescence showing the recruitment of both receptors into endocytic vesicles after 60 minutes of agonist treatment. In this case, cells were permeabilized with 0.2% NP-40 before the immunofluorescence reaction. Nuclei were stained with Hoechst 33258 (1 μg/ml). Scale bars, 10 μm. **C**. β_2_AR-Gα_s_and wild type β_2_AR expressing cells were cultured on 96 multiwell plates and treated with isoproterenol for the indicated times (*abscissae*). Fixed cells were immuno-stained with Flag-antibody followed by Alexa-Fluor 488-conjugated anti-mouse IgG. Nuclei were stained with Hoechst 33258 (1 μg/ml). Fluorescence was measured by a multi-label counter. Each point represents the mean of 4 different experiments performed in triplicate. Data are expressed as ratios of the relative fluorescence measured after immuno-staining with the Alexa-Fluor 488-labeled secondary antibody (RFUFitc) and following Hoechst staining of the same monolayers (RFUHoechst), as described in *Methods*. Data were fitted to an exponential decay equation of the form: y = a_1 _exp (-k t) + a_0_, where t is time in min, a_1 _and a_0 _are decaying and not decaying components of fluorescence, respectively, and k, the time constant, represents the inverse of the half-time (t_1/2_) of the disappearance of immunoreactivity from the cell surface.

The exposure of cell surface β_2_ARs to agonist results in a time-dependent internalization of receptors. Internalization properties of the β_2_AR-Gα_s _fusion protein were investigated using both qualitative and quantitative experimental strategies. Immunofluorescence experiments were performed on HEK293 cells stably expressing the Flag-tagged chimeric (HEK293/β_2_AR-Gα_s_) or wild type (HEK293/β_2_AR) receptors following visualization with an epifluorescence microscope. Figure 2A shows a typical experiment in which the cells were treated with 1 μM isoproterenol for the indicated times, fixed in formaldehyde and subjected to indirect immunofluorescence using an anti-Flag antibody, as described in the *Methods *section. As shown in figure 2A, the wild type receptor rapidly disappeared from the plasma membrane within 5 minutes of agonist treatment. The same amount of time was not sufficient to induce a significant internalization of the chimeric receptor, as indicated by the high immunoreactivity to Flag-antibody retained on the plasma membrane of cells expressing the fusion protein. An appreciable decrease in the surface levels of the fusion protein started at 20 minutes, but the extensive loss of plasma membrane β_2_AR-Gα_s _was only observed after 60 minutes of agonist stimulation. At this time, indeed, the permeabilization of samples with a non-anionic detergent, performed before the immuno-staining procedure, allowed for the observation of an intracellular redistribution of the wild type as well as the chimeric receptor (fig. 2B). To quantify the internalization rates of the chimeric and wild type proteins, we performed a fluorimetric assay. HEK293 cells stably expressing β_2_AR-Gα_s _or native receptor were seeded onto multiwell plates and treated with 1 μM isoproterenol for the indicated times (fig. 2C). After fixation, the surface receptors were immuno-stained with an anti-Flag antibody and labelled with Alexa Fluor 488-conjugated anti mouse IgG. The fluorescence measured by a multi-label counter clearly indicates that the chimeric protein has a lower rate of internalization than the wild type receptor. The computed t_1/2 _was 12 and 33 min for β_2_AR and β_2_AR-Gα_s_, respectively.

### Inefficient recycling of internalized β_2_AR-Gα_s_

**Figure 3:**
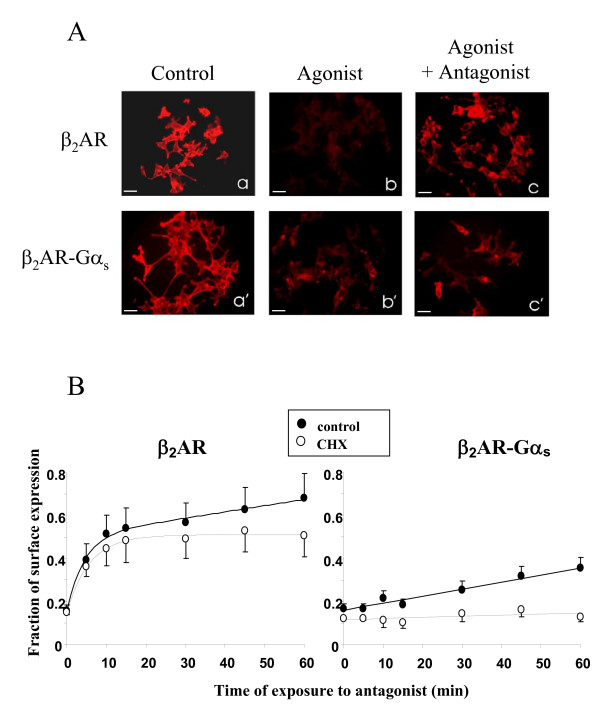
**Antagonist-mediated recycling of internalized β_2_AR andβ_2_AR-Gα_s_**. **A. **Cells stably expressing the β_2_AR or the fusion protein β_2_AR-Gα_s _were treated (panels b, b', c, c') or not (panels a, a') with the agonist isoproterenol to induce complete receptor internalization. The agonist-mediated endocytosis was stopped by the addition of the antagonist propranolol for 60 minutes (panels c,c'). Fixed cells were then processed by fluorescence microscopy for membrane immuno-localization of Flag-tagged receptors, as described in *Methods*. Scale bars, 20 μm. **B**. Cells expressing wild type or fusion protein receptor were incubated with 1 μM isoproterenol for 5 hours to induce maximal internalization. Reversal of internalization was initiated by adding 1 μM propranolol, and cells were fixed and subjected to immuno-staining with Flag-antibody and Alexa Fluor 488-conjugated anti mouse IgG, at the indicated times, to detect the recovery of the cell surface receptors. Data (means of 3 experiments) are reported as the percent of the control (not stimulated) receptor immunoreactivity. The same experiment was performed in the absence (control) or presence of cycloheximide (CHX).

To investigate whether the fusion of β_2_AR to G protein α-subunit also affects the recycling processes of the receptor, we used the same technical approach used for the internalization studies. First, HEK293/β_2_AR or HEK293/β_2_AR-Gα_s _cells were exposed to 1 μM isoproterenol for 5 hours to induce the maximal agonist-mediated internalization, as shown in figure 2C. Then we added the antagonist propranolol and incubated the cells for an additional hour, to allow the cell surface recovery of the receptors. Finally, an immunofluorescence assay was performed to visualize the recycling of epitope-tagged receptors via fluorescence microscopy (fig. 3A). Flag-epitopes immuno-staining revealed that, after 5 hours of incubation with isoproterenol, both receptors disappeared from the cell surface (fig. 3A panels b,b'). However, following an additional hour of incubation with the antagonist propranolol, native β_2 _AR was almost completely returned to the cell surface, in sharp contrast with the β_2_AR-Gα_s _protein, which showed only a slight plasma membrane redistribution (fig. 3A panels c,c'). The inability of the chimeric receptor to recycle back to the plasma membrane was also confirmed by a fluorimetric assay performed to estimate the cell surface recovery of receptors upon antagonist incubation (fig. 3B). HEK293/β_2_AR or HEK293/β_2_AR-Gα_s _cells were cultured on 96 multiwell plates and exposed to isoproterenol (1 μM) for 5 hours. The agonist-mediated recruitment of receptors to intracellular compartments was stopped by the addition of propranolol (1 μM) for increasing times, after which an immunofluorescence assay was performed to visualize the surface receptors (fig. 3B). At each time point, the cell surface recovery of either epitope-tagged β_2 _AR or β_2_AR-Gα_s _was evaluated by the measurement of cell surface fluorescence by a multi-label counter. As shown in figure 3B, upon addition of the antagonist, the wild type receptor rapidly relocalized to the plasma membrane, in a time-dependent fashion. Approximately 50% of internalized β_2_AR returned to the plasma membrane within 15 minutes, thus showing a fast component of recycling. Over this time, the further exposure of cells to antagonist favoured a nearly complete recycling of β_2_AR by a slow component. As expected, the difference in the recycling rates between fused and non-fused receptors was dramatic. In fact, the fluorimetric measurement performed on cells expressing the β_2_AR-Gα_s _resulted in a slow as well as modest relocalization of receptors to the plasma membrane, and no significant recycling of the fusion protein was observed until after 60 minutes of antagonist treatment. Moreover, to exclude the membrane exposure of newly synthesized receptors from analysis, we also performed the same experiments in the presence of cycloheximide (CHX), a protein synthesis inhibitor. Under this condition, we observed that the recovery of the wild type receptor occurred by both recycling (fast component) and resynthesis (slow component). In contrast, the recovery of the fused receptor occurred only by new synthesis.

### β_2_AR-Gα_s _and β_2_AR co-localization with endocytosed transferrin

**Figure 4:**
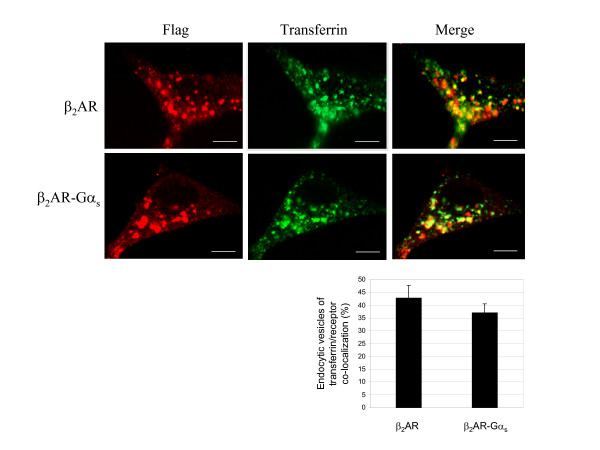
**β_2_AR-Gα_s _and β_2_AR co-localization with endocytosed transferrin**. **A. **The cells were incubated for 30 min with 100 μM Alexa-Fluor 488 conjugated transferrin, a marker of early and recycling endosomes, and with 1 μM isoproterenol for another 30 minutes. The samples were then washed, fixed and immuno-stained with antibody against Flag-epitopes, as described in *Methods*. The sub-cellular distribution and co-localization of both receptors with transferrin were revealed by confocal microscopy. The data are representative images from three independent experiments. *Red*, Flag-tagged receptor Alexa-Fluor 594 stained; *Green*, Alexa-Fluor 488 conjugated transferrin. In the merged images, numerous *yellow *vesicular spots, corresponding to the co-localization between internalized receptors and transferrin, are visualized. Scale bars, 10 μm. **B**. The graph indicates the fraction of endocytic vesicles in which internalized receptors co-localized with the Alexa-Fluor 488 conjugated transferrin. Error bars represents the S.D. of data collected from multiple fields (*n *= 6) from 4 independent experiments.

In order to investigate the failure of the chimeric receptor to recycle back to the plasma membrane in more detail, we compared the agonist-mediated sub-cellular localization of β_2_AR and β_2_AR-Gα_s _receptors with that of endocytosed transferrin, a well-established marker of early and rapid recycling endosomes [14,22,23]. The extent of co-localization between endocytosed transferrin and either native or chimeric receptors was examined by dual-label confocal microscopy (fig. 4A). As indicated by the *yellow *spots in the merged panel of figure 4A, most of the wild type β_2_AR co-localized with the tracer transferrin, consistent with the typical rapid recycling of the β_2_AR, whereas β_2_AR-Gα trafficked through transferrin-positive endosomal compartments, with an apparent lower grade compared to the wild type receptor. The fraction of vesicles showing transferrin-receptor co-localization was approximately 43% and 37% for the wild type and chimeric receptors, respectively (fig. 4B). This slight difference could be explained by the different kinetics of internalization displayed by the two receptors (fig. 2). As the above experiment demonstrated, 30 minutes corresponds to the agonist-exposure time at which β_2 _AR, but not β_2_AR-Gα_s _receptor, shows maximal agonist-mediated internalization. Thus, the resistance to agonist-induced endocytosis showed by the fusion protein could also result in a different kinetic rate of transfer into and out of specific vesicles, without, however, changing its endosomal targeting. Taken together, these data support the hypothesis that the failure of β_2_AR-Gα_s _to undergo recycling is not linked to it sorting into endosomal compartments distinct from those that efficiently translate their cargo to the plasma membrane.

### β_2_AR-Gα_s _and wild type receptor are targeted to the same endocytic vesicles

The experiments reported above do not exclude the possibility that, following agonist-mediated internalization, β_2_AR-Gα_s _and wild type β_2_AR may traffic through similar endosomal compartments. To better understand this, we carried out experiments in which the endocytic trafficking of β_2_-adrenergic receptors was analyzed in a co-expression system using a different tagged version of the native receptor.

**Figure 5:**
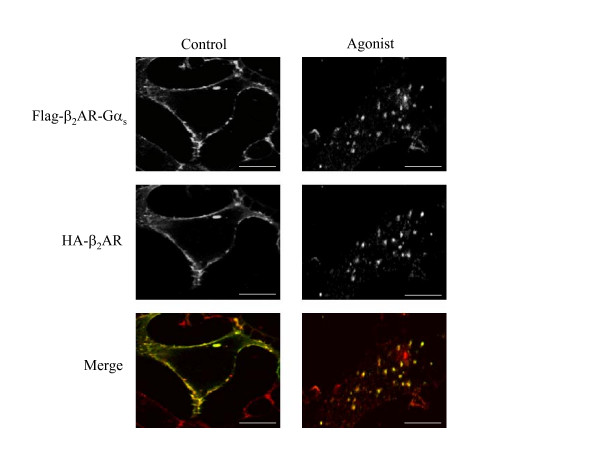
**Dual-localization of co-expressed β_2_-adrenergic receptors by confocal microscopy**. HEK293 cells stably expressing Flag-tagged β_2_AR-Gα_s _receptor were transiently transfected with a HA-tagged β_2_AR construct to obtain the co-expression of the two receptors in the same cell. Cells were treated (Agonist) or not (Control) with 1 μM isoproterenol for 5 hours, fixed and subjected to indirect immunofluorescence analysis as described in *Methods*, and then visualized by confocal microscopy. Co-localization of HA-β_2_AR (Alexa-Fluor 488 staining; *green*) with Flag-β_2_AR-Gα_s _(Alexa-Fluor 594 staining; *red*) is shown in the merged colour image by *yellow *staining. Photographs represent a typical situation observed in 4 independent experiments. Scale bars, 10 μm.

HEK293/β_2_AR-Gα_s _cells were transiently transfected with an amino-terminus HA-tagged β_2_AR (HA-β_2_AR), were incubated with or without 1 μM isoproterenol for 5 hours to induce maximal internalization without affecting the recycling of the native receptor, were fixed, and were then subjected to confocal microscopy. In the absence of agonist stimulation, the immunoreactivity of Flag-β_2_AR-Gα_s_, as well as that HA-β_2_AR, was typically localized at the plasma membrane. In response to agonist, each receptor redistributed from the cell surface to a population of cytoplasmic vesicles that resulted in complete overlapping in the merged colour images (fig. 5). These results, in addition to the transferrin co-localization experiments, confirm that the fusion of the G protein α-subunit to the wild type β_2_AR does not target the chimeric receptor to endosomal compartments distinct from those observed for the wild type protein. Next, using the same experimental strategy, we also examined whether or not the inability of the chimeric receptor to recycle back was affected by the presence of the wild type receptor.

**Figure 6:**
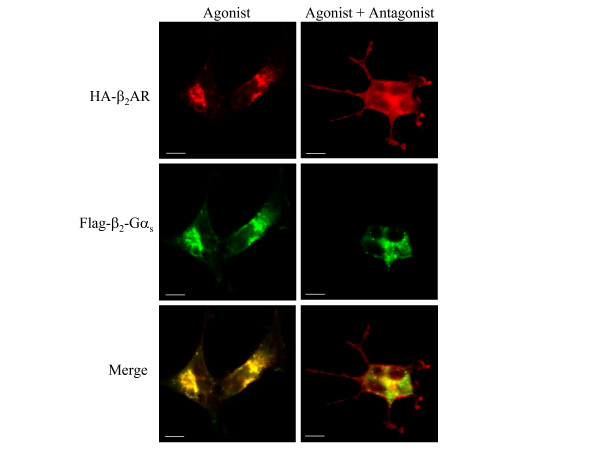
**Dual-localization of co-expressed β_2_-adrenergic receptors after antagonist-mediated recycling**. HEK293 cells stably expressing Flag-β_2_AR-Gα_s _receptor were transiently transfected with HA-β_2_AR construct. After 48 hours, receptor internalization was induced by incubating cells with 1 μM isoproterenol for 5 hours. 1 μM propranolol was added (Agonist + Antagonist) or not (Agonist) for a further 60 minutes to stop receptor endocytosis. Fixed and immuno-stained cells were subjected to epifluorescence microscopy, as described in *Methods*, to visualize the sub-cellular localization of HA-β_2_AR (Alexa-Fluor 594 staining; *red*) and Flag-β_2_AR-Gαs (Alexa-Fluor 488 staining; *green*). Scale bars, 10 μm. Note that the antagonist allows the membrane return of the wild type receptor but not of the chimeric protein. Photographs represent the typical situation observed in 4 independent experiments.

Analysis by epifluorescence microscopy of HEK293/β_2_AR-Gα_s _cells transiently expressing the wild type β_2_AR, and treated for five hours with 1 μM isoproterenol, confirmed that both receptors were recruited into the same endocytic vesicles (fig. 6). However, when the antagonist propranolol was added to stop receptor internalization, we observed an almost complete recovery of the wild type receptor on the cell surface, whereas the β_2_AR-Gα_s _was entirely retained in the intracellular vesicles. Moreover, an intracellular pool of both receptors continued to co-localize, probably in correspondence of compartments that drive them to a common degradative pathway.

## Conclusion

In this study, we have investigated the effect of a C-terminally tethered G protein α-subunit on the recycling rate and post-endocytic fate of the β_2_AR receptor. We observed two important differences between the fused and non-fused receptors.

The first is a substantial reduction in the internalization rate of the receptor-Gα fusion protein compared to the native β_2_-adrenergic receptor (fig. 2). Therefore, depending on the extent to which intracellular recruitment in a given cell contributes to the attenuation of receptor responsiveness, these results suggest that β_2_AR-Gα_s _may be less susceptible to desensitization. This also explains previous reports indicating an increased resistance to desensitization and an enhanced anti-proliferative effect of isoproterenol in S49 cells transfected with this type of fusion protein [24].

The reduced rate of internalization was apparently not due to a divergent process of endocytosis, since β_2_AR-Gα_s _was targeted to the same endosomal compartment where the wild type receptor was translated (fig. 4, 5, 6). This might reflect a reduced ability of the fused receptor to interact with G protein receptor kinases (GRKs), since receptor-Gα fusion proteins were found less capable of interacting with Gβγ subunits, which play a crucial role in recruiting GRKs to the receptor [19]. A detailed study on the ability of β_2_AR-Gα_s _to undergo GRK-mediated phosphorylation and β-arrestin docking will obviously be necessary to address such questions.

The second and more remarkable difference between Gα-fused and non-fused receptor is the total inability of β_2_AR-Gα to undergo recycling, which makes endocytosis an essentially irreversible process for the fusion protein. In fact, the fast CHX-insensitive component of receptor recovery to the cell surface was absent in cell expressing the fused receptor and no recovery of fusion protein was observed in cells treated with CHX (fig. 3), suggesting that fusion proteins coming slowly back to the membrane after internalization are only those derived from *de novo *protein synthesis (fig. 3). Direct labelling in pulse-chase experiments will be necessary to verify such possibility. Moreover, multiplexed immunofluorescence staining of differentially tagged fused and wild type receptors co-transfected in the same cell clearly showed that, unlike native receptors, the fused protein is selectively retained in an intracellular compartment following the reversal of internalization (fig. 6).

It is known that, upon agonist activation, β_2_-adrenergic receptor and the Gα-subunit dissociate and leave the plasma membrane to traffic through distinct endosomal compartments, both of which result in recycling [18]. Moreover, unlike β_2 _AR, Gα_s _does not colocalize with internalized transferrin, indicating that it does not traffic into common recycling endosomes [25].

Thus, it is reasonable to suppose that, in the β_2_AR-Gα construct, the receptor sequence played a critical role in dictating the intracellular trafficking of the fusion protein in response to agonist. On the other hand, the undissociable covalent bond that ties together the two sequences appears to be a sufficient modification to block the natural recycling mechanisms of both proteins. While more investigations will be necessary to identify the exact nature of the endocytic vesicles that irreversibly trap the fusion protein inside the cell, our study allows us to pinpoint which step of receptor endocytosis is most crucially dependent on the physical separation of receptor from Gα. The lack of dissociation, in fact, can reduce the efficiency but does not prevent the initial phase of receptor endocytosis. It does however disrupt the post-endocytic fate of both receptor and Gα subunit.

## Methods

### Materials

Materials came from the following sources: cell culture media, fetal bovine serum, G418 and Lipofectamine were from Invitrogen. Isoproterenol, propranolol, anti-FLAG monoclonal antibody (M1 clone), anti-HA polyclonal antibody and Hoechst 33258 were from Sigma. Phospho-p44/42 MAPK rabbit monoclonal antibody was from Cell Signalling Technology. Alexa-Fluor 488-conjugated transferrin, and antibodies Alexa-Fluor 488 and Alexa-Fluor 594 goat anti-mouse IgG, were from Molecular Probes. Nitrocellulose transfer membrane was from Schleicher&Schuell. Enhanced chemiluminescent substrate (ECL) for detection of HRP was from PIERCE.

### Plasmids

The construction of full-length cDNA encoding the Flag-tagged β2AR-Gαs fusion protein was described previously [19].

The amino terminal Flag tagged wild receptor was obtained in a similar way. Briefly, the cleavable prolactin signal peptide tethered to the Flag epitope (DYKDDDDK) was added immediately before the Gly^2 ^in β_2 _AR by PCR-based strategies and cloned in a pcDNA3 vector (Invitrogen).

The cDNA encoding for 3HA-β_2_AR was obtained by the annealing of two synthetic oligonucleotides containing the sequences of three sequential modules of the influenza hemagglutinin HA epitope (AYPYDVPDYA), and was cloned into pcDNA3 vector. Then, by PCR, we obtained the cDNA encoding for the β_2_AR deprived of the Met initiator codon, which was subcloned into the vector, in frame with the 3 × HA epitope.

### Cell Culture and Transfection

Human embryonic kidney (HEK293) cells stably expressing the wild type β_2_AR or chimeric protein β_2_AR-Gα_s _were generated as described previously [19]. The total number of expressed receptors was measured in membranes prepared from the transfected cells using radioligand binding assays, as described previously [19]. The levels of receptor expression were 15 and 10.8 pmol/mg in cells expressing wild type β_2_AR and β_2_AR-Gα_s_, respectively.

Cells were grown in Dulbecco's modified Eagle's medium (DMEM; GIBCO) supplemented with 10% (v/v) fetal bovine serum (FBS; GIBCO), 100 units/ml penicillin, 100 μg/ml streptomycin sulphate, and 200 μg/ml G418 (GIBCO) in a humidified atmosphere of 5% CO_2 _at 37°C.

For transient transfections, cells cultured in 35-mm tissue culture dishes were transfected with 0.3 μg of pcDNA3/HA-β_2_AR and 0.7 μg of empty vector using Lipofectamine (Invitrogen), according to the manufacturer's instructions. The cells were allowed to express the transfected gene for 48 hrs before harvesting.

### Immunofluorescence

Cells were grown on 35-mm tissue culture dishes and following the various treatments were fixed with 4% buffered formaldehyde for 20 minutes. If necessary, fixed cells were permeabilized with 0.2% Nonidet P-40 (NP-40) in phosphate-buffered saline (PBS) for 10 min to assure the accessibility of intracellular and intravesicular antigens. For the detection of epitope-tagged receptors, anti-Flag mouse monoclonal antibody was added in blocking buffer (1% BSA) for 50 min at room temperature (r.t.) followed by Alexa-Fluor 594 conjugated anti-mouse IgG (Molecular Probes). In dual staining experiments, co-localization of Flag-tagged receptors and HA-tagged receptors in a single cell line, was performed by incubating cells with anti-Flag and anti-HA rabbit polyclonal antibody (Sigma).

Stained specimens were examined by conventional epifluorescence microscope (Olympus BX51; Tokyo, Japan) or confocal microscope.

### Confocal Laser Scanning Microscopy

Fluorescently labelled preparations were also observed by a confocal fluorescent imaging system using the confocal laser scanning microscope LEICA TCS 4D (Leach Instruments, Heidelberg, Germany) supplemented with an Argon/Kripton laser and equipped with 40 × 1.00–0.5 and 100 × 1.3–0.6 oil immersion lenses. The excitation/emission wavelengths employed were 488 nm/510 nm, and 568 nm/590 nm for specific Alexa-Fluor labelling. Confocal sections were acquired at intervals of 0.5 μm from the middle to the bottom toward the cells, and a 3D reconstruction image of the fluorescent signal was obtained. In double staining experiments, confocal sections for both fluorescent signals were taken simultaneously, the 3D reconstruction images were recorded, and merged images of the two signals were obtained using confocal microscope software.

### Receptor Internalization Assays

Cells, cultured on 96 multiwell plates (Nunc) until they reached confluence, were stimulated with 1 μM isoproterenol (Sigma) at 37°C for different times to drive agonist-induced internalization. At the end of the time-course, cells were immediately fixed with 4% buffered formaldehyde for 20 min, incubated in blocking buffer for 30 min and immuno-stained for 50 min with anti-FLAG mouse monoclonal antibody (Sigma), followed by Alexa-Fluor 488 conjugated anti-mouse IgG (Molecular Probes). Nuclei were stained with Hoechst 33258 (1 μg/ml). Fluorescence was measured by a multi-label counter (Victor 2; Wallac), setting excitation and emission filters as follows: λ_ex _= 485/λ_em _= 535, and λ_ex _= 355/λ_em _= 460 for Alexa-Fluor 488 and Hoechst, respectively. Each point was performed in triplicate and the receptor internalization was monitored in relationship to the decrease of fluorescence from the plasma membrane.

### Receptor Recycling Assays

For recycling studies, cells cultured on 96 multiwell plates were incubated with 1 μM isoproterenol for 5 hrs to induce maximal receptor internalization. Reversal of internalization was initiated by adding the antagonist, 1 μM Propranolol (Sigma), for different times. In other experiments, 50 μg/ml cycloheximide (CHX; Sigma) was added during the last 2 hrs of agonist treatment. At the end of the time-course, the cells were fixed and subjected to immunofluorence followed by fluorimetry analysis as above.

Receptor recycling from the endocytic pathway was estimated by assaying the recovery of immunoreactive epitopes at the cell surface that were accessible by monoclonal antibody.

### Localization of Receptor with Endocytosed Transferrin

In order to estimate the degree of co-localization of the receptor with endocytosed transferrin, cells were incubated for 30 min at 37°C in culture medium containing 100 μM Alexa-Fluor 488-conjugated transferrin (Molecular Probes), and then 1 μM isoproterenol was added for an additional 30 min.

Next, cells were washed twice in pre-warmed PBS to remove non-internalized transferrin and were subjected to immunofluorescence as above. Briefly, fixed cells were permeabilized and immuno-stained for detection of Flag-tagged receptors. Dual-label fluorescence microscopy was performed as described above.

### Immunoblotting for Phospho p42/44 MAP Kinases

Cells were first sensitized by incubating with the agonist isoproterenol (1 μM) for different times and were then dissolved in Laemmli's sample buffer. Samples were electrophoresed through a 12% SDS-polyacrylamide gel (SDS-PAGE) and transferred onto nitrocellulose membranes (Schleicher&Schuell). Blots were incubated in blocking buffer (5% w/v non fat dry-milk, 0,1% Tween-20 in Tris buffered saline) for 1 hrs at r.t., followed by incubation overnight at 4°C with Phospho-p44/42 MAP kinases rabbit monoclonal antibody (Cell Signalling Technology), which detects endogenous levels of p42 and p44 MAP kinases (ERK1/2) when phosphorylated at Thr202 and Tyr204, respectively. Immunoreactive bands probed with horseradish peroxidase-conjugated anti rabbit IgG antibody were visualized by chemiluminescence (ECL; PIERCE), according to the manufacturer's instructions.

Samples were normalized for protein content by reprobing blots with an alpha-tubulin monoclonal antibody (Sigma).

## Competing interests

The authors declare that they have no competing interests.

## Authors' contributions

MGDC designed experiments and wrote the manuscript. In addition, MGDC performed Receptor Internalization/Recycling Assays. EB performed cell culture, transfection, immunoblotting and immunofluorescence experiments. IC constructed the vector encoding for 3HA-β_2_AR. AS analyzed the confocal experiments. AF and CP contributed reagents/materials/analysis tools. PM and EM supervised and provided guidance and critical input into the study. All authors have read and approved the final version of the manuscript.
